# FDA-Approved Natural Polymers for Fast Dissolving Tablets

**DOI:** 10.1155/2014/952970

**Published:** 2014-04-27

**Authors:** Md Tausif Alam, Nayyar Parvez, Pramod Kumar Sharma

**Affiliations:** Department of Pharmacy, School of Medical and Allied Sciences, Galgotias University, Plot No. 2, Sector 17-A, Yamuna Expressway, Gautam Buddh Nagar, Greater Noida, Uttar Pradesh 201306, India

## Abstract

Oral route is the most preferred route for administration of different drugs because it is regarded as safest, most convenient, and economical route. Fast disintegrating tablets are very popular nowadays as they get dissolved or facilely disintegrated in mouth within few seconds of administration without the need of water. The disadvantages of conventional dosage form, especially dysphagia (arduousness in swallowing), in pediatric and geriatric patients have been overcome by fast dissolving tablets. Natural materials have advantages over synthetic ones since they are chemically inert, non-toxic, less expensive, biodegradable and widely available. Natural polymers like locust bean gum, banana powder, mango peel pectin, *Mangifera indica* gum, and *Hibiscus rosa-sinenses* mucilage ameliorate the properties of tablet and utilized as binder, diluent, and superdisintegrants increase the solubility of poorly water soluble drug, decrease the disintegration time, and provide nutritional supplement. Natural polymers are obtained from the natural origin and they are cost efficacious, nontoxic, biodegradable, eco-friendly, devoid of any side effect, renewable, and provide nutritional supplement. It is proved from the studies that natural polymers are more safe and efficacious than the synthetic polymers. The aim of the present article is to study the FDA-approved natural polymers utilized in fast dissolving tablets.

## 1. Introduction

Of all the dosage forms administered orally, the tablet is one of the most preferred dosage forms. Disintegrants are agents integrated to tablet and some encapsulated formulations to promote the breakup of the tablet and capsule “slugs” into more small fragments in an aqueous environment thereby incrementing the available surface area and promoting a more rapid release of the drug substance. They promote moisture penetration and dispersion of the tablet matrix. Tablet disintegration has received considerable attention as an essential step in obtaining fast drug release. The accentuation on the availability of drug highlights the importance of the relatively rapid disintegration of a tablet as a criterion for ascertaining uninhibited drug dissolution behavior. Number of factors affects the disintegration replace of tablets. The disintegrants have the major function to oppose the efficiency of the tablet binder and the physical forces that act under compression to compose the tablet. The more strong the binder, the more efficacious must be the disintegrating agents in order for the tablet to release its medication. Ideally, it should cause the tablet to disrupt, not only into the granules from which it was compressed, but additionally into powder particles from which the granulation was yare. Disintegrants are an essential component to tablet formulations. The ability to interact strongly with water is essential to disintegrate function. Combination of swelling and/or wicking and/or deformation are the mechanisms of disintegrant action. A disintegrant utilized in granulated formulation processes can be more efficacious if utilized both “intragranularly” and “extragranularly” thereby acting to break the tablet up into granules and having the granules further disintegrate to release the drug substance into solution. However, the portion of disintegrant integrated intragranularly (in wet granulation processes) is conventionally not as efficacious as that integrated extragranularly due to the fact that it is exposed to wetting and drying (as a component of the granulation process), which reduces the activity of the disintegrant. Since a compaction process does not involve its exposure to wetting and drying, the disintegrant used intragranularly inclines to retain good disintegration activity [1, 2]. The polymers obtained from the natural inchoation are more efficacious and safe. They are facilely available in natural regions around the world therefore they are preferred over synthetic polymer. Natural polymers are utilized in most of the preparation and are more propitious over synthetic polymers as they are economical, and they have low cost and are facilely available in the sufficient quantity. Natural polymers are nontoxic; they do not have any adverse effects on the body. Natural polymers are environmental friendly as they are biodegradable in nature they do not cause any pollution. Natural polymers are devoid of side effects as they are obtained from the natural source. Natural polymers are mainly preferred by the patients as they are more safe and efficacious as compared to the synthetic polymers and have more patient compliance. Natural polymers provide nutritional supplement and are renewable as they are utilized again and again in different reactions [3].

## 2. Ideal Properties of Fast-Dissolving Tablets

- They should be disintegrated within seconds when placed in mouth.
- They should not require water to dissolve.
- Being unit dosage forms, they should provide precise dosing.
- Quick dissolution and absorption in the oral cavity.
- Easy to convey.
- Tablets are manufactured with conventional equipment with low cost.
- Less sensitive to environmental condition like humidity and temperature.
- They should be less fragile and should maintain its hardness [4].

## 3. Advantages of Fast Dissolving Tablet

- Fast dissolving tablets (FDTs) are solid unit dosage form, so they provide precise dosing, and high drug loading is sanctioned in it, and it is an ideal dosage in case of geriatric and pediatric patients, and additionally it is an ideal alternative of conventional tablet.
- It has fast action, as it is taken by the patient, it commences melting when it comes in contact with saliva, it rapidly absorbed in the oral cavity, and it rapidly melts and produces fast action.
- Due to pregastric absorption, the bioavailability of the drugs is amended, and fewer doses are required, which amends the patient compliance, clinical reports are also amended.
- Fast dissolving tablets do not require water to swallow, and also they can be taken anywhere at any time, and they are a convenient option for travelling patients and diligent peoples who do not have immediate access of water; hence, patient compliance is amended.
- They are very facile and convenient to administer as they are a solid unit dosage form, and they are mainly convenient for geriatric, pediatric, uncooperative patients and dysphagic patients.
- Fast dissolving tablets are very safe and facile to swallow because there is no peril of suffocation in the airways due to physical obstruction during swallowing.
- Fast dissolving tablets contain minimal leaves, and they thoroughly dissolve in the mouth; no residue is left, so they provide good mouth feeling and hence improved palatability of the tablet.
- Fast dissolving tablets are less sensitive to environmental condition; hence, they are very stable.
- Fast dissolving tablets are packed in simple blister packaging, and there is no need of special and costly packaging, so they are economical.
- Fast dissolving tablets provide incipient business avenues as product differentiation, product promotion, line extension, uniqueness, and life cycle management.
- Fast dissolving tablets are cost efficacious; they do not require costly ingredients. Natural polymers, when utilized as excipient, are available facilely and at low cost, and withal they do not require special packaging material, they can be packed in simple blister packs.
- They are a multifarious technology, as they are utilized in the development of over-the-counter (OTC) medicines, Rx medicines, and veterinary medicines.
- They are facilely portable as they are a solid dosage form and less sensitive to environmental condition and do not require water to swallow the dosage form [5, 6].

## 4. Natural Polymers Used in Fast Dissolving Tablets

The utilization of natural polymers is valuable predicated on proven biocompatibility and safety. Natural gums are among the most popular hydrophilic polymers because of their cost-efficacy and regulatory acceptance. Polymers are generally employed in floating drug distribution systems so as to target the distribution of drug to a concrete region in the gastrointestinal tract, that is, stomach. Moreover, these polymers are safe, nontoxic, and capable of chemical modification and gel formation [7]. See Table 1.

## 5. Advantages of Natural Polymers

The various advantages of natural plant based materials include the following.

- *Biodegradable: *Biodegradable as they are naturally available, and they are produced by all living organisms.
- *Biocompatible and non-toxic:* Basically, all of these plant materials are reiterating sugar polysaccharides.
- *Low cost:* They are cheaper to utilize as natural sources. The production cost is less compared with the synthetic material. India and many other developing countries are dependent on agriculture, and there are substantial amounts of money investment on agricultures.
- *Environmental-friendly processing:* There are many types of natural compounds obtained from different plant sources which are widely utilized in pharmaceutical industry and collected in immensely large quantities due to the simple production processes involved.
- *Local availability (especially in developing countries):* In India and homogeneous developing countries, there is promotion for the production of plants as pharmaceutical excipients being done by government, and it withal provides the facilities for bulk production, like gum and mucilage's because of their wide applications in industries.
- *Patient tolerance as well as public acceptance:* There is less chance of side and adverse effects with natural materials compared with synthetic one [8].

## 6. Classification of Polymers Used in the Fast Dissolving Tablet

1. Natural polymer
2. Synthetic polymer
3. Semi-synthetic polymer

*Natural Polymer*. These are various plant based materials. Plant-based material serves as an alternative to synthetic products because of following reasons:

- Local accessibility
- Eco-friendliness
- Bio-acceptability
- Having renewable source and low price as compared to synthetic products

## 7. Natural Polymers Used in Fast Dissolving Tablets

### 7.1. Chitin and Chitosan

Chitin (*β*-(1→4)-N-acetyl-D-glucosamine) is a natural polysaccharide obtained from crab and shrimp shells. It possesses amino group covalently linked to acetyl group as compared to liberate amino group in chitosan. Chitosan is produced commercially by deacetylation of chitin, which is the structural element in the exoskeleton of crustaceans (such as crabs and shrimp) and cell walls of fungi. Bruscato and Danti, 1978, reported that when chitin was included in the conventional tablets, the tablets disintegrated within 5 to 10 minutes irrespective of solubility of the drug. The disintegration time in the oral cavity as well as wetting time could be analyzed by surface free energy. Chitosan is the best kenned natural polysaccharide utilized for its multifarious applications in pharmaceutical industry [9].

### 7.2. Guar Gum

Guar gum is mainly consisting of the high molecular weight (approximately 50,000–8,000,000) polysaccharides composed of galactomannans and is obtained from the endosperm of the seed of the guar plant,* Cyamopsis tetragonoloba* (L) Taub. (Syn.* Cyamopsis psoralioides*). It is utilized as thickener, stabilizer, and emulsifier and approved in most areas of the world (e.g. EU, USA, Japan, and Australia). It is naturally occurring gum (marketed under the trade name Jaguar). It is free flowing, consummately soluble, neutral polymer composed of sugar units and is approved for use in food. It is not sensitive to pH, moisture contents, or solubility of the tablet matrix. It is not always pristine white and sometimes varies in color from off-white to tan and inclines to discolor with time in alkaline tablets [10].

### 7.3. Gum Karaya

Gum karaya is a vegetable gum produced as an exudate by trees of the genus* Sterculia*. Chemically, gum karaya is an acid polysaccharide composed of the sugars galactose, rhamnose, and galacturonic acid. The high viscosity nature of gum limits its uses as binder and disintegrant in the development of conventional dosage form. Gum karaya has been investigated for its potential as a tablet disintegrant. Different results showed that modified gum karaya produces rapid disintegration of tablets. Gum karaya can be utilized as an alternative superdisintegrant to commonly available synthetic and semisynthetic superdisintegrants due to its low cost, biocompatibility as well as facile availability [11].

### 7.4. Agar and Treated Agar

It is the dried gelatinous substance obtained from* Gelidium amansii* (Gelidanceae) and several other species of red algae like* Gracilaria *(Gracilariaceae) and* Pterocladia* (Gelidaceae). Agar is yellowish-gray or white to proximately colorless, inodorate with mucilaginous taste and is available in the form of divests, sheet flakes, or coarse powder. Agar consists of two polysaccharides, agarose and agar pectin. Agarose is responsible for gel vigor and agar pectin is responsible for the viscosity of agar solutions. High gel vigor of agar makes it a potential candidate as a disintegrants [12].

### 7.5. Fenugreek Seed Mucilage

*Trigonella foenum-graceum* commonly kenned as fenugreek, is an herbaceous plant of the leguminous family. Fenugreek seeds contain a high percentage of mucilage (a natural gummy substance present in the coatings of many seeds). Albeit it does not dissolve in water, mucilage forms a viscous tacky mass when exposed to fluids. Like other mucilage-containing substances, fenugreek seeds swell up and become slick when they are exposed to fluids. Hence, the study revealed that this natural disintegrant (fenugreek mucilage) showed more preponderant disintegrating property than the most widely used synthetic superdisintegrants like Ac-di-sol in the formulations of FDTs. Studies betokened that the extracted mucilage is a good pharmaceutical adjuvant and concretely a disintegrating agent [13].

### 7.6. Soy Polysaccharide

It is a natural superdisintegrants that does not contain any starch or sugar so can be utilized in nutritional products. Halakatti et al. 2010 [14] evaluated soy polysaccharide (a group of high molecular weight polysaccharides obtained from soy beans) as a disintegrant in tablets made by direct compression utilizing lactose and dicalcium phosphate dihydrate as fillers. A cross-linked sodium carboxymethyl cellulose and corn starch were utilized as control disintegrants. Soy polysaccharide performs well as a disintegrating agent in direct compression formulations with results paralleling those of cross-linked CMC [15, 16].

### 7.7. Gellan Gum

Gellan gum is a water-soluble polysaccharide produced by* Pseudomonas elodea*, a bacterium. Gellan gum is an anionic, high molecular weight, deacetylated exocellular polysaccharide gum produced as a fermentation product by a pristine culture of* Pseudomonas elodea* with a tetra saccharide reiterating unit of one *α*-L-rhamnose, one *β*-D-glucuronic acid, and two *β*-D-glucose residues. Antony and Sanghavi 1997 studied the gellan gum as a disintegrant and the efficiency of gum was compared with other conventional disintegrants such as dried corn starch, Explotab, Avicel (pH 10.2), Ac-di-sol, and Kollidon CL. The disintegration of tablet might be due to the instantaneous swelling characteristics of Gellan gum when it comes in contact with water and owing to its high hydrophilic nature. The consummate disintegration of tablet was has proved itself as superior disintegrant [16].

### 7.8. Mango Peel Pectin

Mango peel which constitutes 20–25% of the mango processing waste was found to be a good source for the extraction of pectin of good quality, felicitous for the preparation of film, and acceptable jelly. Pectin is an in volute heteropolysaccharide which is a hydrophilic colloid. Malviya et al. (2011) investigated and found that mango peel pectin stands as a good candidate as superdisintegrant, though not as more strong than synthetic superdisintegrants, but due to its good solubility and higher swelling index, it may be utilized in the formulation of fast dispersible tablets [17, 18].

### 7.9. *Lepidium sativum* Mucilage

*Lepidium sativum* (family: Cruciferae) is kenned as Asaliyo and is widely utilized as herbal medicine in India. It is widely available in market and has very low cost. Components used are leaves, root, oil, seeds, and so forth. Seeds contain higher amount of mucilage, dimeric imidazole alkaloids lepidine B, C, D, E, and F, and two incipient monomeric imidazole alkaloids, semilepidinoside A and B. Mucilage of* Lepidium sativum* has different characteristics like binding, disintegrating, gelling, and so forth [19].

### 7.10. *Plantago ovata* Seed Mucilage

Psyllium or ispaghula is the prevalent name utilized for several members of the plant genus* Plantago *whose seeds are utilized commercially for the production of mucilage. Mucilage of* Plantago ovata* has different characteristics like binding, disintegrating, and sustaining properties. In an investigation fast disintegrating tablets of amlodipine besylate was yare by direct compression method utilizing different concentrations of* Plantago ovata* mucilage as natural superdisintegrants. All formulations were evaluated for weight variation, hardness, friability, disintegration time, drug content, and dissolution. The optimized formulation shows less* in vitro* disintegration time of 11.69 seconds with rapid* in vitro* dissolution within 16 minutes.* In vitro* disintegration time decreases with increase in concentration of natural superdisintegrant [20, 21].

### 7.11. *Aegle marmelos* Gum (AMG)

It is obtained from the fruits of* Aegle marmelos* belonging to the disintegrated faster and consistently than the croscarmellose sodium. The ripened fruit pulp is red in color with mucilaginous and astringent taste. The pulp contains carbohydrates, proteins, vitamin C, vitamin A, angelenine, marmeline, dictamine, O-methyl fordinol and isopentyl halfordinol. AMG is prepared by heat treatment technique. It increases the solubility of poorly soluble drugs. It increases glucose level and glycosylated hemoglobin in diabetic patients, decreases plasma insulin and liver glycogen in diabetic patient, decreases lipid peroxidation, stimulates macrophage functioning, and causes significant deviation in the GSH (glutathione) concentration in liver, kidney, stomach, and intestine. Purified, bael gum polysaccharide contains D-galactose (71%), D-galacturonic acid (7%), L-Rhamnose (6.5%), and L-arabinose (12.5%) [22].

### 7.12. Locust Bean Gum

It is known as carob bean gum. It is a galactomannan vegetable gum extracted from the seeds of carob tree (*Ceretonia siliqual*) found in Mediterranean region. Locust bean gum is utilized as gelling and thickening agent in food industry and utilized as bio adhesive, and it enhances the solubility. The gum is a white to yellowish-white, odorless powder. It is insoluble in most organic solvents including ethanol. It is partially soluble in water at ambient temperature and soluble in hot water and needs heating to above 850 for 10 min for complete solubility [23].

### 7.13. Ficus Indica Fruit Mucilage

The mucilage of ficus indica fruit is utilized as superdisintegrant which is obtained from the pulp of fruit ficus indica. Ficus indica is an astronomically immense tree up to 3 meters and very fast-growing with spread branches and arial roots. The fruits of ficus indica are of the size of cherry. It has nutritional as well as medicinal value. The dried and uncooked ficus indica fruit gives 230 kcal (963 KJ) of energy per 100 gm or 3.5 oz. (ounce). It is utilized in assuaging fever, pain, inflammation, wound rejuvenating, blood quandaries, and urinary quandaries [24].

### 7.14. *Mangifera indica* Gum (MIG)

Mundane name of* Mangifera indica *is mango, and it belongs to Anacardiaceae family. It is nontoxic and utilized as disintegrant, binder, suspending agent, and emulsifying agent in different formulations. The gum powder is white to off white in colour, and the powder was soluble in water and virtually insoluble in acetone chloroform, ether, methanol, and ethanol. It is facilely available, and gum is devoid of toxicity, and each and every component of the tree has pharmacological activity like diuretic, astringent, diabetes, asthma, diarrhea, urethritis, and scabies [25].

### 7.15. Hibiscus Rosa Sinensis Mucilage and Treated Agar

It is withal called shoe flower plant, China rose, and Chinese hibiscus and belongs to the family Malvaceae. Mucilages are utilized as thickeners, suspending agent, water retention agent, and disintegrants. The plant is facilely available and its leaves contain mucilage and is present in mucilage L-rhamnose, D-galactose, D-galacturonic acid, and D-glucuronic acid. Treated agar is yare by treating it with water for one day [14].

### 7.16. Dehydrated Banana Powder (DBP)

Banana is additionally called plantain. DBP is yare from the variety of banana called Ethan and nenthran (*nenthra vazha*) and belongs to the family Musaceae. It contains vitamin A, so it is utilized in the treatment of gastric ulcer and diarrhea. It withal contains vitamin B6, which avails in reducing the stress and solicitousness. It is a very good source of energy due to high carbohydrate content, and it contains potassium, which is responsible for more preponderant brain functioning [26].

## 8. Current Regulatory Status of These Polymers

All these Polymers are approved by US Food and Drug Administration (FDA). The FDA recognizes these polymers as GRAS (Generally Recognized as Safe), as listed in the Code of Federal Regulations (CFR 21), for example, chitosan, guar gum, Locust and bean gum. Gum karaya fully meets all specifications as outlined in the Food Chemicals Codex and may be safely used in foods as described in the Federal Register (21 CFR).

Gellan gum is approved as a food additive in the European community under the number E 418, with ADI (acceptable daily intake) confirming its status as a safe food additive. The Gellan gum food grade fully meets the standards and the purity criteria issued in different regions of the world or internationally, such as the Food Chemicals Codex and JECFA, the US Pharmacopoeia/National Formulary, and the European Directives. So, these polymers are safe and can be safely used.

## 9. Conclusion

Natural polymers have more preponderant effects on fast dissolving tablets than synthetic polymers. Natural polymers incremented the drug release rate from the tablet and decremented the dissolution and disintegration time, and they are utilized as binder superdisintegrant and diluent. Natural polymers are preferred over synthetic polymers as they are nontoxic, facilely available at low cost, utilized in low concentration, and are naturally extracted to provide nutritional supplement. The disintegrating properties of* Plantago ovata*,* Lepidium sativum*,* gum karaya, Guar gum*,* Fenugreek seed mucilage, mango peel pectin*, and so forth, have been studied in comparison to artificial super disintegrants. Thus natural superdisintegrants exhibit faster drug dissolution and increased bioavailability, thereby, availing in efficacious therapy and improved patient compliance. Thus the natural superdisintegrant can be efficaciously utilized as disintegrants in tablet formulations.

## Figures and Tables

**Table 1:** Natural polymers used in fast dissolving tablets.

S. no.	Natural polymer	Marketed drug	Disintegration time	Concentration used
1	Chitin and chitosan	Cinnarizine	60 sec	3% w/w
2	Guar gum	Glipizide	30 sec	1% w/w
3	Gum karaya	Amlodipine, granisetron hydrochloride	17.10 sec	4% w/w
4	Agar and treated agar	Theophylline	20 sec	1-2% w/w
5	Fenugreek seed mucilage	Metformin hydrochloride	15.6 sec	4% w/w
6	Soy polysaccharide	Lornoxicam	12 sec	8% w/w
7	Gellan gum	Metronidazole	155 sec	4% w/w
8	Mango peel pectin	Aceclofenac	11.59 sec	0.1–4% w/w
9	*Lepidium sativum* mucilage	Nimesulide	17 sec	5–15% w/w
10	*Plantago ovata* seed mucilage	Granisetron HCl	17.10 sec	5% w/w
11	Aegle marmelos gum	Aceclofenac	8–18 min	6% w/w
12	Locust bean gum	Nimesulide	13 sec	10% w/w
13	*Lepidium sativum *	Nimesulide	17 sec	10% w/w
14	*Mangifera indica* gum	Metformin HCL, paracetamol	3–8 min	6% w/w
15	*Hibiscus rosa-sinensis* mucilage	Aceclofenac	20 sec	6% w/w
16	Dehydrated banana powder	Ondansetron HCl/propranolol, gabapentin	15–36 sec	6% w/w
